# Molecular Genetic Analysis of the Rabies Virus in Wild and Domestic Animals of Kazakhstan and the Development of a Diagnostic System

**DOI:** 10.3390/pathogens15090886

**Published:** 2026-08-24

**Authors:** Kulyaisan T. Sultankulova, Aigerim K. Abubakirova, Gaukhar O. Shynybekova, Meirim D. Almezhanova, Nurlan S. Kozhabergenov, Zhumagali K. Koshemetov, Kamshat A. Shorayeva, Kuandyk D. Zhugunissov, Mukhit B. Orynbayev

**Affiliations:** 1 Research Institute for Biological Safety Problems, National Holding QazBioPharm, The Ministry of Health Care of the Republic of Kazakhstan, Gvardeiskiy 080409, Kazakhstan; sh.gauhar@biosafety.kz (G.O.S.); m.almezhanova@biosafety.kz (M.D.A.); n.kozhabergenov@biosafety.kz (N.S.K.); zh.koshemetov@biosafety.kz (Z.K.K.); k.shorayeva@biosafety.kz (K.A.S.); k.zhugunissov@biosafety.kz (K.D.Z.); omb65@mail.ru (M.B.O.); 2 MVA Group Research and Production Center, Almaty Region, Karasai District, Koksai village 040900, Kazakhstan; 3 Department of Microbiology, Virology and Immunology, Faculty of Veterinary Science and Zooengineering, Kazakh National Agrarian Research University, Ministry of Agriculture of the Republic of Kazakhstan, Almaty 050010, Kazakhstan

**Keywords:** wild and domestic animals, rabies virus, N gene, cosmopolitan, real-time RT-PCR

## Abstract

Rabies remains one of the most significant zoonotic infections, characterized by high mortality. In Kazakhstan, the virus circulates among both domestic and wild animals, necessitating ongoing molecular genetic monitoring and improved laboratory diagnostic methods. The aim of this study was to investigate the genetic characteristics of the rabies virus circulating among animals in Kazakhstan using nucleoprotein (N) gene analysis and to develop a regionally adapted real-time RT-PCR method for its detection. Kazakhstani rabies virus isolates deposited in GenBank revealed that all belong to a cosmopolitan lineage and form a single phylogenetic cluster, regardless of host species. Isolates from neighboring countries, including China and Russia, were also included in this cluster, demonstrating the regional relationship of circulating virus variants. The obtained data were used to develop a real-time PCR method based on the genetic characteristics of the rabies virus circulating in Kazakhstan. The diagnostic performance of the method was evaluated using 144 samples of various origins obtained from domestic and wild animals, including bats. The developed assay demonstrated a sensitivity of 86.4%, a specificity of 98.4%, and a diagnostic efficiency of 96.5%. The proposed method ensures reliable detection of rabies virus variants circulating in Kazakhstan and can be used for laboratory diagnostics and epidemiological monitoring.

## 1. Introduction

Kazakhstan is located in the center of the Eurasian continent at the junction of two continents—Europe (15% of the territory) and Asia (85% of the territory)—and occupies a central, connecting, transit position. The epidemiological situation regarding rabies in Kazakhstan has remained tense throughout the entire period of official registration. This pattern is characteristic not only of Kazakhstan but also of many European and Asian countries [1,2]. According to WHO data, approximately 60,000 people die from rabies annually, predominantly in Asian and African countries, which underscores the high social and economic significance of the disease [3].

The causative agent is the rabies virus of the genus Lyssavirus, family Rhabdoviridae, which exhibits pronounced neurotropism and the ability to be transmitted among various mammalian species [4]. The main reservoirs of the virus are wild and domestic carnivores; however, other species may be involved in the epizootic process, which determines the complex nature of pathogen circulation.

The global rabies eradication program within the framework of the ‘One Health’ concept, adopted in 2018, provides many countries with opportunities to achieve a final victory over this disease [5,6,7].

The Cosmopolitan lineage of rabies virus is widely distributed across Eurasia, including Kazakhstan [8], Russia [9], and northwestern regions of China [10,11], as well as the autonomous region of Inner Mongolia [12]. It is characterized by a high degree of genetic homogeneity and stable interspecies circulation among various animal species.

The spread of strains in this group across a vast geographic range indicates prolonged, stable, and epizootically significant transboundary circulation of the rabies virus [13].

Both wild animals (foxes, jackals, bats) and domestic animals (cats, dogs, cattle) are involved in the epizootic process, forming a unified epizootic cycle. It has been established that the virus is freely transmitted between species without significant genetic adaptation to a specific host, which substantially complicates disease control and increases the risk of its spread at the transboundary level [14].

In recent years, the development of highly sensitive and specific molecular diagnostic methods enabling rapid and reliable detection of rabies virus has become of particular importance. The effectiveness of such methods depends on the selection of conserved genomic regions suitable for the design of universal primers and probes. Within the framework of epidemiological surveillance and testing of domestic and wild animals, these tests will be used, especially in central reference laboratories.

In current practice, molecular genetic methods play a key role in rabies diagnostics, particularly real-time RT-PCR, which provides high sensitivity and specificity of analysis. International guidelines for rabies diagnostics developed by the World Organisation for Animal Health (WOAH) emphasize the need for highly accurate methods of laboratory confirmation of infection [15].

One of the key challenges in molecular rabies diagnostics is the selection of conserved genomic regions of the virus. The nucleoprotein gene (N) is the most suitable target owing to its high degree of conservation among various rabies virus strains and is widely used in the development of diagnostic test systems [16].

The rabies virus continues to circulate in the Republic of Kazakhstan among domestic and wild animals, necessitating the continuous improvement of laboratory diagnostic methods. One area of focus is the development of molecular diagnostic test systems using sequences of the virus circulating in a specific region. This approach allows for the consideration of the genetic diversity of local isolates and the creation of regionally adapted diagnostic methods designed to detect the rabies virus in the Republic of Kazakhstan.

The objective of this study was a comparative molecular genetic analysis of Kazakhstani rabies virus isolates and the development of a regionally adapted real-time RT-PCR test system based on the N gene for the detection of rabies virus RNA circulating in the Republic of Kazakhstan.

## 2. Materials and Methods

### 2.1. Biological Samples

To evaluate the diagnostic performance of the developed real-time RT-PCR assay, 144 samples of various origins were analyzed, including samples from bats, as well as samples containing rabies virus RNA from domestic and wild animals. Biological samples from animals with clinical signs of disease were collected by state veterinarians as part of routine epidemiological surveillance. Samples were submitted for testing to the Laboratory for Monitoring of Viral and Bacterial Infections of the Research Institute for Biological Safety Problems (RIBSP) from regional laboratories of the Republic of Kazakhstan. Brain tissue samples collected from deceased animals were placed separately in plastic bags labeled with the animal species and tissue number. Prior to delivery to the laboratory, the material was stored at −20 °C, and transportation was carried out in a thermos with ice. Detailed information on the sample collection site and animal species is shown in Table 1.

### 2.2. Viral RNA/DNA Extraction

Nucleic acids were extracted from biological samples containing rabies virus, as well as from heterologous viruses used to evaluate the analytical specificity of the developed real-time RT-PCR assay. RNA was extracted from preparations of rabies virus strain “CVS”, Canine distemper virus strain “LD”, Peste des petits ruminants virus strain “G-45”, and Bluetongue virus strain “BTV Targap”. DNA was extracted from Aujeszky’s disease virus strain “UB-95”. All heterologous viruses originated from the virus strain collection of the Research Institute for Biological Safety Problems.

Extraction of viral nucleic acids was performed using the PureLink Viral RNA/DNA Mini Kit (Invitrogen, Thermo Fisher Scientific, Carlsbad, CA, USA) according to the manufacturer’s instructions. Nucleic acid concentration was determined using a NanoDrop 2000 spectrophotometer (Thermo Fisher Scientific, Waltham, MA, USA). The purity of the isolated nucleic acids was assessed by the A260/A280 optical density ratio.

### 2.3. Nucleotide Sequences of Kazakhstani Rabies Virus Isolates Used for Primer and Probe Design

Nucleotide sequences of the nucleoprotein (N) gene of Kazakhstani rabies virus isolates deposited in the GenBank database (NCBI) were used for the design of primers and a fluorescent probe. The analysis included 38 sequences obtained from various animal species (cattle, dog, cat, fox, jackal, and bat): OP477393, OP477390, OP477388, OP477387, OP477386, OP477385, OP477384, OP477383, OP477382, OP477379, OP477378, OP477377, OP477376, OP477375, OP477374, OP477373, OP477372, OP477370, OP477369, OP585396, KT965737, KT965735, KT965734, KT965733, OP477392, OP477391, OP477389, OP477381, OP477380, OP477371, OP477368, ON366710, ON366709, ON366708, ON366707, ON366706, KT965738, and KT965736. These sequences were used for multiple sequence alignment to identify conserved regions of the N gene and the subsequent design of primers and a fluorescent probe for the real-time RT-PCR system.

### 2.4. Design of Primers and Fluorescent Probe for Real-Time RT-PCR

For the development of the real-time RT-PCR assay, the N gene of rabies virus, containing conserved regions suitable for highly specific molecular diagnostics, was selected as the molecular target. The analysis used nucleotide sequences of 38 Kazakhstani rabies virus isolates previously deposited in GenBank. Multiple sequence alignment was performed using MEGA 11 software version 11, which made it possible to assess the degree of conservation of the N gene and identify variable regions.

Analysis of the nucleotide sequences showed a high degree of conservation of the studied N gene region among all included isolates, regardless of host species and geographic origin. Based on the alignment results, conserved regions were selected for the design of specific primers and a fluorescent probe. When designing the oligonucleotides, melting temperature (Tm), GC content, absence of potential secondary structures, and the presence of nucleotide substitutions in the putative binding sites were taken into account. The specificity of the designed oligonucleotides was assessed using the BLAST + version 2.17.0. The low level of variability and the absence of multiple substitutions within the primer- and probe-annealing regions indicated the stability of the selected N gene regions and allowed their use for the development of the real-time RT-PCR assay for the detection of rabies virus. Primers and fluorescent probes were designed using Vector NTI Advance 11.5.

**Figure 1:**
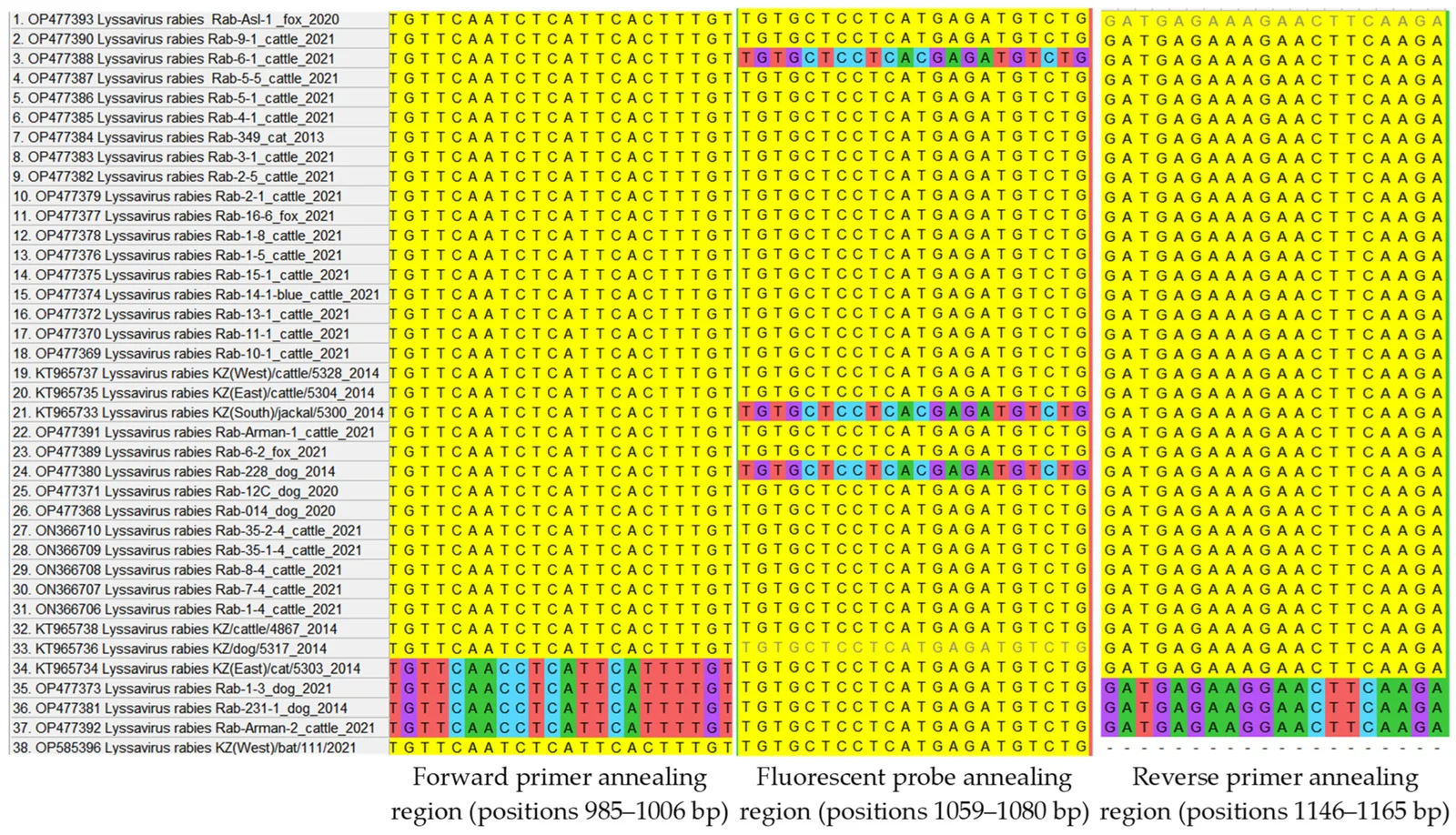
Schematic location of the forward primer, fluorescent probe, and reverse primer binding sites within the rabies virus N gene. The diagram was constructed based on multiple alignment of nucleotide sequences of 38 Kazakhstani rabies virus isolates performed in MEGA 11.

The schematic location of the primers and fluorescent probe within the structure of the N gene, obtained based on multiple sequence alignment in MEGA 11, is shown in Figure 1.

The binding-site coordinates of the primers and fluorescent probe are given relative to the sequence of the Kazakhstani isolate Rab-Asl-1 (GenBank: OP477393), used as the reference for multiple alignment. The forward primer N-Rab_F corresponds to positions 985–1006, the fluorescent probe RN Probe to positions 1059–1080, and the reverse primer N-Rab_R to positions 1146–1165 relative to this sequence. The length of the amplified fragment is 180 bp.

Alignment analysis showed that the identified nucleotide substitutions were limited in nature and generally did not exceed 1–2 positions within the analyzed regions. Two variable positions were identified in the forward primer annealing region—992 (T→C) and 1001 (C→T). To compensate for genetic variability, degenerate bases were introduced into the forward primer sequence according to IUPAC nomenclature (Y = T/C).

One variable position was found in the reverse primer binding region—1154 (G→A). Since this substitution is located outside the critical 3′ end of the primer and does not significantly affect amplification efficiency, a degenerate base was not used.

One variable position was identified in the fluorescent probe hybridization region θ 1070 (T→C); to compensate for it, a degenerate base Y = T/C was introduced at the corresponding position of the probe.

The sequences and main characteristics of primers N-Rab_F and N-Rab_R, as well as the fluorescent probe RN Probe, are presented in Table 2.

### 2.5. Real-Time RT-PCR Assay Procedure

Amplification was performed in a one-step real-time RT-PCR format using reverse transcriptase and Taq DNA polymerase (Eurogen, Moscow, Russia). The total reaction volume was 25 µL and included: 5 µL of 5× reaction buffer, 1.25 µL of MgCl_2_ (50 mM; final concentration 2.5 mM), 0.5 µL of dNTP mix (2.5 mM each dNTP; final concentration 50 µM each dNTP), 1.0 µL each of forward N-RabF and reverse N-RabR primers (10 µM; final concentration 400 nM each primer), 1.0 µL of fluorescent probe RN Probe (5 µM; final concentration 200 nM), 0.25 µL of Taq DNA polymerase (1.25 U), 0.5 µL of reverse transcriptase (40 U), the test RNA, and nuclease-free water to a final volume of 25 µL. Amplification was carried out under the following conditions: reverse transcription at 50 °C for 30 min, initial denaturation at 94 °C for 15 min, followed by 40 cycles consisting of denaturation at 94 °C for 15 s and annealing/elongation at 55 °C for 30 s. Fluorescence signal detection was performed in the FAM channel. Recombinant plasmid pJET1.2/blunt Cloning Vector (Thermo Fisher Scientific Baltics UAB, Vilnius, Lithuania), containing a 180 bp insert of the rabies virus N gene fragment, was used as a positive control. Nuclease-free water served as the negative control. Results were interpreted based on the threshold cycle (Ct) value. Samples with a Ct value ≤ 35 were considered positive for rabies virus RNA, whereas samples with a Ct value > 35 or with no amplification signal were classified as negative.

### 2.6. Validation of the Developed Real-Time RT-PCR Assay for Rabies Virus Detection

#### 2.6.1. Analytical Sensitivity

To determine the analytical sensitivity of the developed real-time RT-PCR assay, a series of ten-fold dilutions of pJET1.2 plasmid DNA containing a 180 bp insert of the rabies virus N gene fragment, with concentrations ranging from 10 ng to 10 ag, was tested in the reaction mixture. Each dilution was tested in triplicate. Using plasmid DNA allows us to calculate the copy number of the target sequence and estimate the detection limit during the PCR amplification step. The limit of detection was defined as the minimum concentration of the positive control material at which amplification was recorded in all replicates with a Ct value ≤ 35.

#### 2.6.2. Analytical Specificity

The specificity of the developed primers and fluorescent probe was initially assessed in silico using the BLAST program (NCBI) by comparing their nucleotide sequences with sequences available in the GenBank database. The analytical specificity of the developed real-time RT-PCR assay was experimentally evaluated using heterologous viruses: Canine distemper virus, Aujeszky’s disease virus, Peste des petits ruminants virus, and Bluetongue virus. This panel was selected because these viruses are epizootiologically significant causative agents of infectious diseases in domestic and wild animals, are diagnosed in veterinary laboratories, and may be present in the test material during differential diagnosis. Specificity was considered confirmed in the absence of amplification for all tested heterologous viruses.

#### 2.6.3. Diagnostic Sensitivity and Specificity

The diagnostic sensitivity and specificity of the developed real-time RT-PCR assay were evaluated by comparing the results with the reference method—rabies virus isolation [17]. A total of 144 biological samples were included in the study. According to the results of the reference virus isolation method, 22 samples were classified as positive for rabies virus and 122 as negative. Positive samples were obtained from cats (n = 3), cattle (n = 13), dogs (n = 3), jackals (n = 2), and one common noctule (*Nyctalus noctula*, n = 1). All other tested samples were negative. Samples in which rabies virus was confirmed by virus isolation were classified as true positive (TP), while samples with a negative virus isolation result were considered true negative (TN). Samples with a positive real-time RT-PCR result and a negative virus isolation result were considered false positive (FP), and samples with a negative real-time RT-PCR result and a positive virus isolation result were considered false negative (FN).

When determining the performance characteristics of the laboratory test, calculations were based on the following formulas: sensitivity (SN) = TP/(TP + FN), specificity (SP) = TN/(TN + FP), positive predictive value (PPV) = TP/(TP + FP), negative predictive value (NPV) = TN/(TN + FN), diagnostic efficiency (DE) = (TP + TN)/(TP + FP + FN + TN). The 95% confidence interval (95% CI) was estimated using the Wilson score method [18].

#### 2.6.4. Repeatability

Repeatability (intra-assay reproducibility) was assessed by testing the positive control sample three times within a single experiment under identical reaction conditions. For each replicate, the threshold cycle (Ct) value was determined, after which the mean Ct value, standard deviation (SD), and coefficient of variation (CV, %) were calculated.

#### 2.6.5. Reproducibility

Reproducibility was assessed by repeated testing of the positive control sample in three independent real-time RT-PCR runs. To assess variability, the mean Ct value, standard deviation, and coefficient of variation were calculated. The repeatability of the developed real-time RT-PCR assay was assessed by testing the same positive control sample in three technical replicates within a single run. Inter-assay reproducibility was assessed by repeated testing of the same positive control sample in three independent real-time RT-PCR runs performed on different days. For each run, the mean Ct value, standard deviation (SD), and coefficient of variation (CV, %) were calculated [19].

### 2.7. PCR Amplification of the Rabies Virus Nucleoprotein Gene

Amplification of the PCR product of the rabies virus nucleoprotein gene was performed using the SuperScript III Platinum One-Step Quantitative RT-PCR System (Invitrogen, USA) according to the manufacturer’s instructions. The primer pair Rab-NF—ATGGATGCCGACAAGATTGT and Rab-NR—GCACACTGTTGTTCAACTCC was used to amplify the PCR product of the rabies virus nucleoprotein gene. The size of the PCR product was 1372 bp.

### 2.8. Sequencing Assays, BLASTn Analysis and Phylogenetic Analyses

Sequencing was performed on an Applied Biosystems 3130 automated DNA sequencer (Hitachi, Tokyo, Japan). The sequencing reaction was performed directly on the PCR products using the BigDye™ Terminator v3.1 Cycle Sequencing Kit (Applied Biosystems, Inc., Vilnius, Lithuania) according to the manufacturer’s instructions. After the sequencing reaction, the products were purified using the BigDye™ XTerminator™ Purification Kit (Applied Biosystems™, Thermo Fisher Scientific, Waltham, MA, USA). Sequencing was performed using the forward and reverse primers employed for amplification. The resulting nucleotide sequences were analyzed in Sequencher v. 4.5 (Gene Codes Corporation, Ann Arbor, MI, USA).

The identities and similarities of the sequenced isolates were analyzed using the Basic Local Alignment Search Tool (BLASTn) of the National Center for Biotechnology Information (NCBI) GenBank database and MEGA 11.0 software [20].

For phylogenetic analysis, available nucleotide sequences of rabies virus strains from the GenBank database were used: AB570997, JX987744, KC595280, KC595282, KJ748633, KT965733, KT965734, KT965735, KT965736, KT965737, KT965738, KU041696, KX148127, KX533959, KY002895, KY765901, MG996454, MK124738, MK124744, MT079918, ON366706, ON366707, ON366708, OP328385, OP477368, OP477371, OP477373, OP477374, OP477377, OP477390, OP477391, OP477384, OP477389, OP477380, OP477381, OP477392, OP477393, OP585396, OQ603640, PX845666, KX148191, KX148186, MK760691, KY860605, KX148181, KX148171, MK760703, KX148176, PZ227540, and KY087943.

Six rabies virus-positive samples were selected for molecular genetic analysis and included in this study. The samples were obtained in 2014 from various animal species in different regions of the Republic of Kazakhstan. After viral RNA isolation and amplification of the target N gene fragment, the PCR products were sequenced at the Research Institute for Biological Safety Problems. The obtained nucleotide sequences were deposited in the GenBank database under accession numbers KZ/(EastKZ)/cat/5303 (KT965734), KZ/(WestKZ)/cattle/5328 (KT965737), KZ/(EastKZ)/cattle/5304 (KT965735), KZ/(Almaty)/cattle/4867 (KT965738), KZ/(Aktobe)/dog/5317 (KT965736), and KZ/(SouthKZ)/jackal/5300 (KT965733) and were used in the present study for phylogenetic analysis, as well as in the development of primers and a fluorescent probe. Additional sequences used for the comparative analysis were obtained from the publicly available GenBank database.

Multiple alignment of nucleotide sequences was performed using the ClustalW algorithm in MEGA11. The phylogenetic tree based on nucleotide sequences of the rabies virus N gene was constructed using the Maximum Likelihood method with the Tamura–Nei substitution model [21]. Initial trees for the heuristic search were automatically generated using the Neighbor-Joining and BioNJ algorithms, after which the topology with the highest log-likelihood value was selected. The reliability of the internal tree branches was assessed by the bootstrap method using 1000 replications. The phylogenetic analysis included 50 nucleotide sequences.

### 2.9. Illustrations

The schematic diagram was prepared using BioRender (https://www.biorender.com/, accessed on 15 June 2026) based on the obtained phylogenetic and molecular data.

## 3. Results

### 3.1. Analytical and Diagnostic Characteristics of the Developed Real-Time RT-PCR Assay for the Detection of Rabies Virus

#### 3.1.1. Determination of Analytical Sensitivity and Specificity

**Figure 2:**
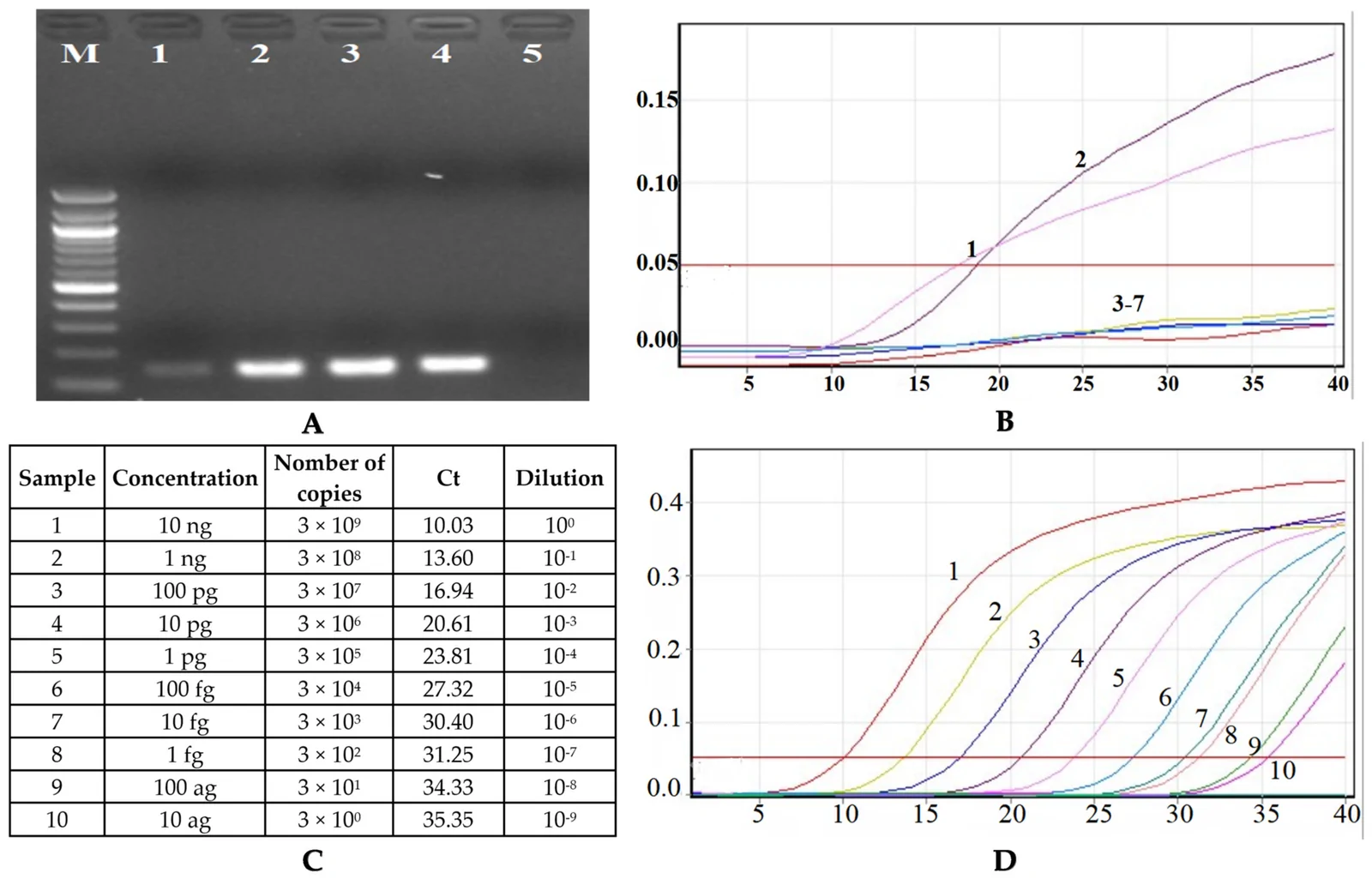
Analytical characteristics of the developed real-time RT-PCR assay for the detection of rabies virus. (**A**): PCR—Lane M: 1 kb DNA ladder (Invitrogen). Lanes 1–3: amplification products from rabies virus-positive samples (expected amplicon size, 180 bp); lane 4: amplification product from the rabies virus-positive control; lane 5: negative control. (**B**): real-time RT-PCR specificity testing—lanes 1, 2: rabies virus RNA (positive control); lanes 3–6: RNA/DNA from heterologous viruses; lane 7: negative control. (**C**): Ct values at different concentrations and serial dilutions. (**D**): 1—10 ng (10^0^); 2—1 ng (10^−1^); 3—100 pg (10^−2^); 4—10 pg (10^−3^); 5—1 pg (10^−4^); 6—100 fg (10^−5^); 7—10 fg (10^−6^); 8—1 fg (10^−7^); 9—100 ag (10^−8^); 10—10 ag (10^−9^).

The diagnostic primers N-Rab_F and N-Rab_R, developed in this study, amplified a 180 bp DNA fragment (Figure 2A).

The analytical sensitivity of the developed real-time RT-PCR assay was evaluated using ten-fold dilutions of plasmid DNA containing a fragment of the rabies virus N gene. Amplification was detected across the entire range of tested concentrations, from 10 ng to 10 ag. Threshold cycle (Ct) values increased consistently as the template concentration decreased—from 10.03 at 10 ng to 35.35 at 10 ag (Figure 2C,D). Amplification was consistently detected at the minimum concentration of 10 ag, which corresponds to the limit of detection of the developed real-time RT-PCR assay. This limit of detection, 10 ag of recombinant plasmid DNA, corresponds to an estimated 3 copies per reaction. The coefficient of determination was R^2^ = 0.99, and the amplification efficiency was 96.53%.

In silico analysis using GenBank and BLAST showed that the developed primers and fluorescent probe are highly specific to rabies virus. No significant homology with sequences from other microorganisms was found.

The specificity of the developed real-time RT-PCR assay was also tested experimentally, using heterologous viruses: Canine distemper virus, Aujeszky’s disease virus, Peste des petits ruminants virus, and Bluetongue virus. Amplification occurred only for rabies virus. None of the heterologous samples produced a signal. This confirms the specificity of the method (Figure 2B).

#### 3.1.2. Assessment of Diagnostic Sensitivity and Specificity

The diagnostic performance of the developed real-time RT-PCR assay was evaluated by comparing its results with virus isolation, used as the reference method. Sensitivity, specificity, positive and negative predictive value, and overall diagnostic efficiency were calculated for a set of 144 samples. The results are shown in Table 3.

The diagnostic sensitivity of the assay was 86.4% (95% CI: 66.7–95.3%). This shows that the method correctly identifies most true-positive samples. Specificity reached 98.4% (95% CI: 94.2–99.5%). This indicates high accuracy in identifying true-negative samples and a low probability of false-positive results. The positive predictive value (PPV) was 90.5% (95% CI: 71.1–97.3%). This means a positive real-time RT-PCR result is highly likely to reflect a true rabies virus infection. The negative predictive value (NPV) was 97.6% (95% CI: 93.1–99.2%). This indicates a high probability that a negative result truly reflects the absence of infection. The overall diagnostic efficiency of the assay was 96.5%. This indicates a high level of agreement between real-time RT-PCR results and virus isolation data. These results demonstrate the strong diagnostic value of the developed real-time RT-PCR assay and confirm that it can be used for the laboratory diagnosis of rabies virus infection.

#### 3.1.3. Assessment of Repeatability and Reproducibility

The repeatability and reproducibility of the developed real-time RT-PCR assay were assessed. The results showed stable performance (Table 4).

When rabies virus RNA was tested three times within a single analytical run, Ct values showed low variability. The mean Ct value was 22.19 ± 0.59, and the coefficient of variation (CV) was 2.67%. When the same sample was tested in three independent analytical runs, the mean Ct value was 22.45 ± 0.19, and the CV was 0.83%. These results indicate high repeatability and reproducibility of the developed real-time RT-PCR assay.

### 3.2. Detection of Rabies Virus in the Tested Samples by Real-Time RT-PCR

A total of 144 samples of pathological material from domestic and wild animals were tested by real-time RT-PCR. Specific rabies virus RNA was detected in 22 samples. This corresponds to 15.3% of all samples tested (Table 5).

Among bats, a positive result was found only in one common noctule (*Nyctalus noctula*). No viral RNA was detected in the particolored bat (*Vespertilio murinus*), serotine bat (*Eptesicus serotinus*), or lesser mouse-eared bat (*Myotis blythii*). Among domestic and wild animals, the highest proportion of positive results was found in cattle (13 of 17 samples; 76.5%). This was followed by dogs (3 of 5; 60.0%), cats (3 of 6; 50.0%), and jackals (2 of 6; 33.3%). Positive samples were found in the West Kazakhstan, Abai, Almaty, Aktobe, and Turkestan regions (Table 6).

**Figure 3:**
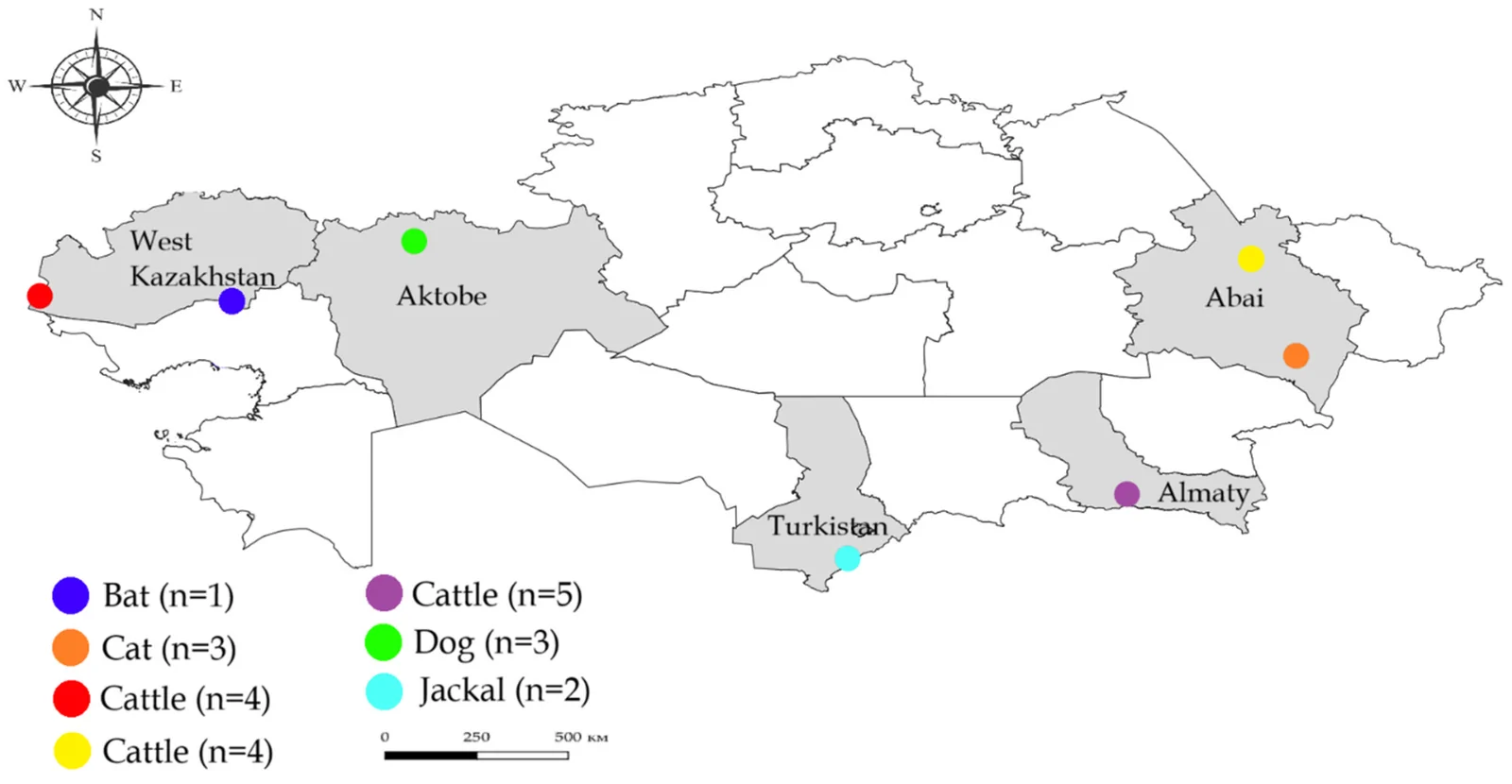
Geographic distribution of the rabies virus samples included in the study across the regions of Kazakhstan. Cartographic visualization was performed in QGIS 3.40 software using OpenStreetMap data.

The largest number of positive samples was found in the Abai region (7 of 16 tested samples). This was followed by the West Kazakhstan region (5 of 18) and the Almaty region (5 of 13). In the Aktobe and Turkestan regions, positive results were found in 3 of 18 and 2 of 24 samples, respectively. In the Zhambyl, Kyzylorda, Atyrau, and North Kazakhstan regions, all tested samples were real-time RT-PCR negative. The map shows the geographic distribution of the 22 rabies virus samples obtained from different animal species across Kazakhstan (Figure 3).

In the West Kazakhstan region, five positive samples were recorded: one from a common noctule and four from cattle. In the Aktobe region, rabies virus RNA was detected in three samples from dogs. In the Turkestan region, a positive result was obtained in two samples from jackals. In the Almaty region, five positive samples were found in cattle. In the Abai region, seven positive samples were recorded, including three from cats and four from cattle.

### 3.3. Phylogenetic Analysis of Rabies Virus N Gene Sequences in Domestic and Wild Animals

**Figure 4:**
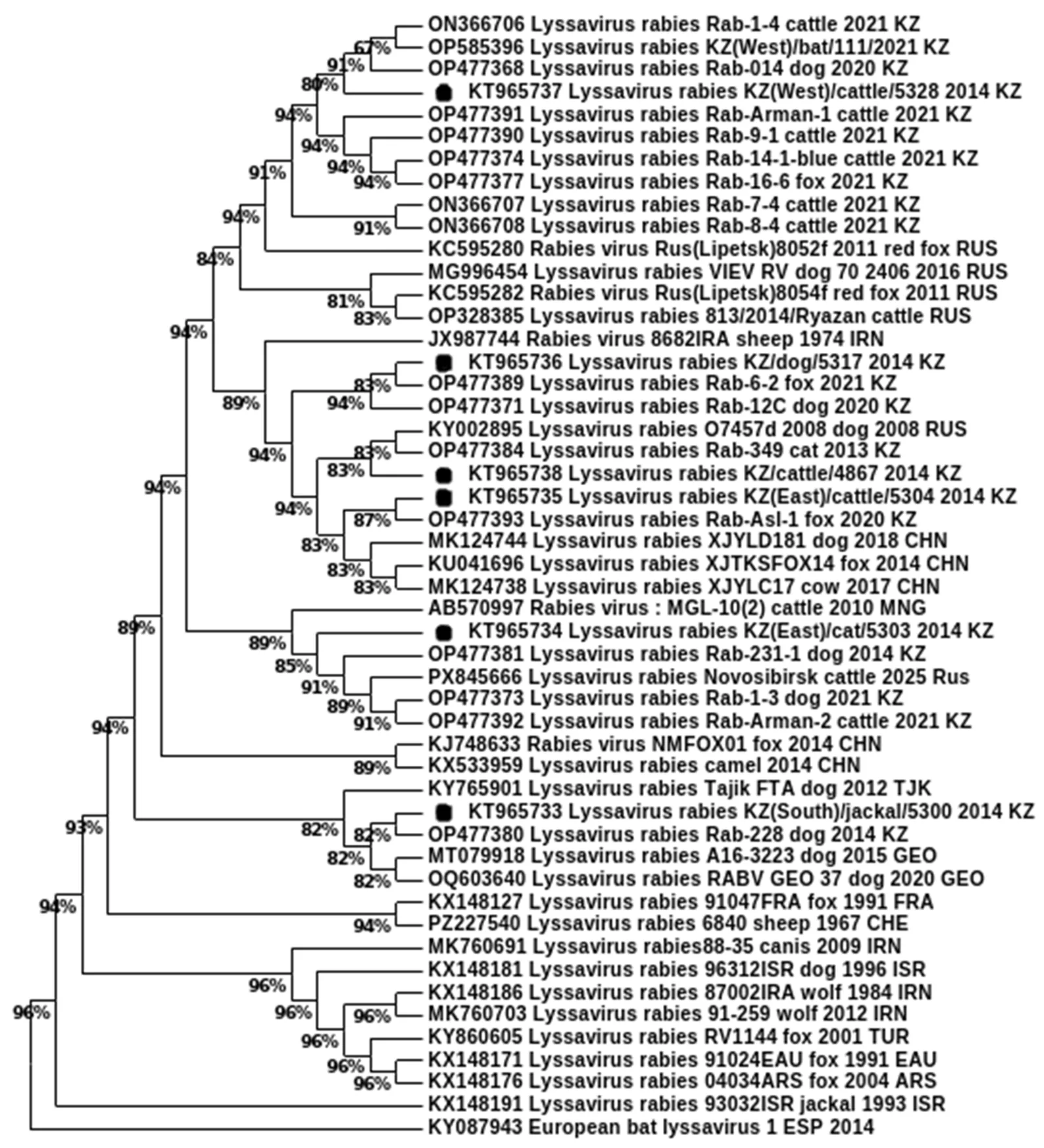
Phylogenetic tree of rabies virus, constructed using the maximum likelihood method, based on nucleotide sequences of the N gene. The Kazakhstani rabies virus isolates investigated in this study are indicated by black circles (●) on the phylogenetic tree.

To determine the phylogenetic relationships between the isolates, a maximum likelihood tree was constructed based on nucleotide sequences of the N gene (Figure 4).

Phylogenetic analysis of the N gene nucleotide sequences showed that all studied rabies virus isolates form a single monophyletic group. This group is defined relative to the outgroup, European bat lyssavirus 1, used to root the tree. All Kazakhstani isolates fell into several well-supported phylogenetic clusters, with bootstrap support values of 81–96%. Kazakhstani isolates grouped together with isolates from Russia (RUS), China (CHN), Mongolia (MNG), Tajikistan (TJK), and Georgia (GEO). They did not form separate clusters based on country of origin. This pattern reflects the circulation of genetically related virus variants in Central Asia and is consistent with their classification within the Cosmopolitan Central Asia lineage. A separate phylogenetic group within the Cosmopolitan clade was formed by isolates from France (FRA) and Switzerland (CHE) belonging to the European group (Cosmopolitan Europe). Another group included isolates from Israel (ISR), Iran (IRN), Turkey (TUR), the United Arab Emirates (EAU), and Saudi Arabia (ARS) corresponding to the Cosmopolitan Middle East group.

Six Kazakhstani rabies virus isolates were previously deposited in GenBank, from cattle (KT965734, KT965735, KT965737, KT965738), a dog (KT965736), and a jackal (KT965733). These isolates were obtained by our research group at the Research Institute for Biological Safety Problems in 2014 and are marked with black circles on the phylogenetic tree. They are distributed across several phylogenetic clusters. All six isolates belong to the Cosmopolitan Central Asia lineage and do not form a separate branch. Although these six isolates were deposited in GenBank in 2014, their phylogenetic analysis had not been reported until now.

### 3.4. Circulation of Rabies Virus Among Wild and Domestic Animals in Kazakhstan

**Figure 5:**
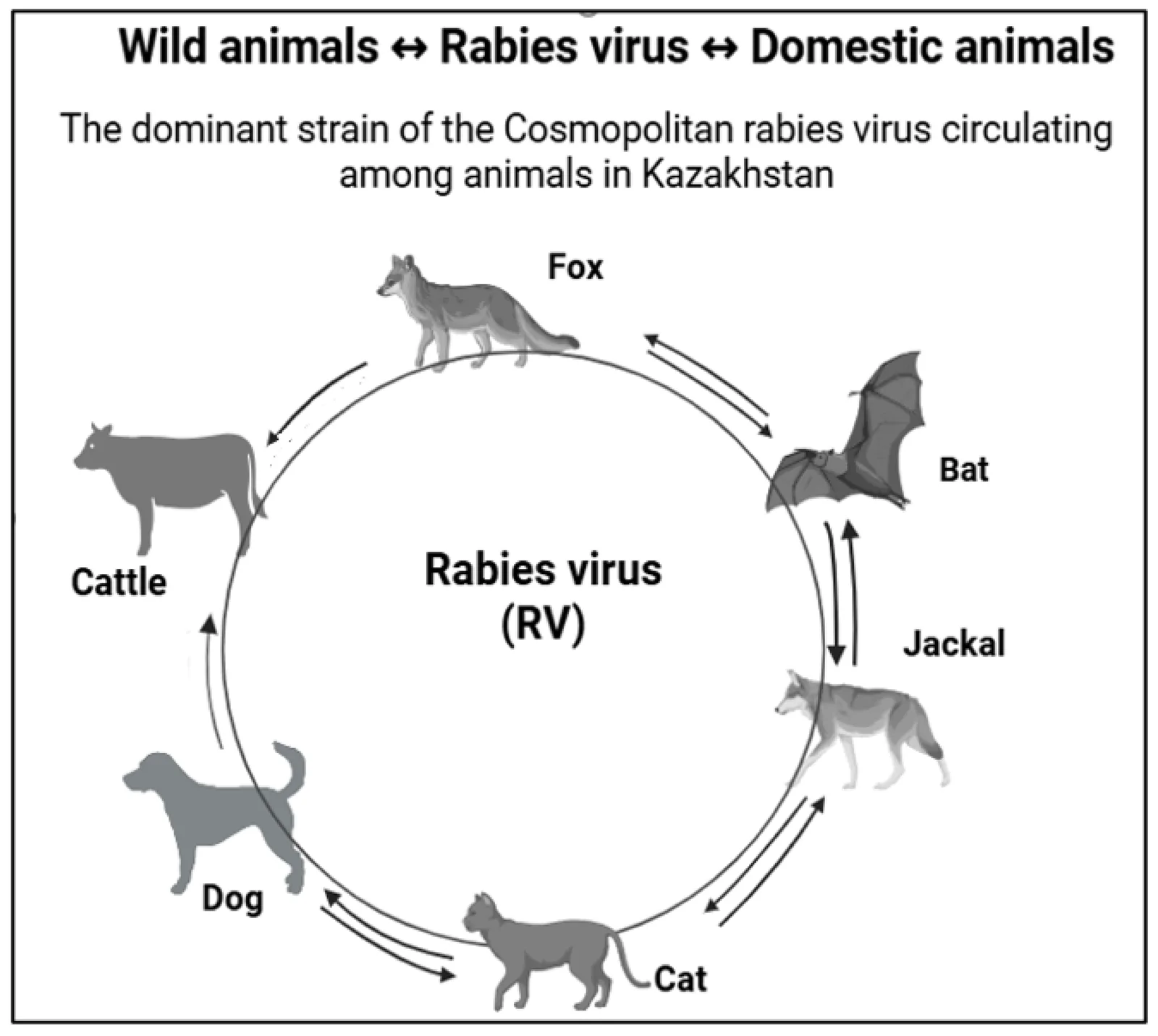
Circulation of rabies virus among wild and domestic animals, driven by the dominant Cosmopolitan lineage in Kazakhstan. The diagram was created using BioRender (BioRender.com).

To visually illustrate the circulation of rabies virus among wild and domestic animals in Kazakhstan, a diagram was developed. It shows the spread of the dominant Cosmopolitan lineage across different host species (Figure 5).

The diagram illustrates the circulation of rabies virus among wild and domestic animals in Kazakhstan. Phylogenetic analysis showed that all studied isolates belong to the Cosmopolitan lineage and share a high degree of genetic similarity, regardless of animal species. No substantial differences were found between viruses isolated from different hosts.

## 4. Discussion

In Eurasia and Asian countries, rabies virus circulates among various animal species. This has resulted in a complex phylogeographic structure. In Europe, viral evolution has involved a limited number of host shifts, including the transition from dogs to foxes and adaptation to raccoon dogs [22,23,24]. In contrast, Asia and Central Eurasia show greater reservoir diversity and more complex patterns of interspecies virus transmission [14].

Domestic dogs served as the original reservoir for modern Eurasian virus lineages. The virus subsequently underwent independent adaptations to various wild carnivore species, including foxes, jackals, and raccoon dogs. This led to the formation of stable natural infection foci. A distinct phylogenetic group has been described for the steppe zone of Eurasia. It circulates mainly among foxes and other wild carnivores, reflecting the virus’s adaptation to specific ecosystem conditions [14]. In East Asia, Arctic-like virus variants have become widespread. They circulate among domestic and wild animals in Russia, Mongolia, and China, including the Inner Mongolia Autonomous Region. This demonstrates the virus’s ability to establish stable reservoir systems in new host populations [22,25]. In addition, independent adaptation events to new host species, such as ferret badgers, have been described. These events are accompanied by the accumulation of species-specific mutations [14].

Spatiotemporal analysis confirms the uneven distribution of rabies and the presence of local epizootic foci in Kazakhstan, including border regions. This indicates active virus circulation and cross-border spread [1]. Taken together, these data highlight the complexity of the epizootic situation and the need for a comprehensive approach to disease monitoring.

The results of this study showed that Kazakhstani rabies virus isolates predominantly belong to the Cosmopolitan lineage. This is consistent with previously published data on the dominance of this lineage in the region [8,26]. Isolates obtained from different animal species form a single phylogenetic cluster, without clear species-specific differentiation. This indicates unrestricted interspecies transmission of the virus. Joint clustering of Kazakhstani, Russian, and Chinese isolates, together with the absence of clear geographic separation, may reflect the cross-border circulation of genetically related rabies virus variants within Central Eurasia. Phylogenetic proximity of Kazakhstani isolates to strains from Russia, China, and other Central Asian countries such as Tajikistan and Georgia indicates a common origin. It confirms the existence of a single cross-border virus pool within the Eurasian region. The absence of clear geographic clustering further indicates long-term, stable virus circulation across large territories.

Molecular genetic methods for rabies diagnosis are widely used in practice worldwide, owing to their high sensitivity and their ability to detect the virus even in degraded samples. Conserved regions of the nucleoprotein (N) gene are among the most commonly used RT-PCR targets. They enable detection of a broad range of rabies virus variants and other lyssaviruses [16].

Previously developed RT-PCR assays have shown high efficiency for diagnosing rabies in various animal species, including cases involving decomposed pathological material, where the sensitivity of the standard FAT test is markedly reduced [27]. Real-time RT-PCR methods have also been shown to have high reproducibility. They are suitable for routine laboratory diagnosis and epizootiological monitoring of the infection [28].

Although several published studies describe real-time RT-PCR assays for the detection of rabies virus, most of them were developed using a limited set of sequences specific to particular geographic regions.

Study [29] proposed an assay comprising three independent sets of primers and probes targeting different regions of the N gene (nucleotides 55–165, 630–781, and 846–1020). It was designed to improve the universality of detection across different genetic variants of rabies virus. In [30] the analysis was based on amplification of a region of the N gene spanning nucleotides 707–899. This assay was validated mainly on African rabies virus isolates, reflecting the regional focus of that assay.

In contrast to previously published studies, the development of primers and a fluorescent probe in this study used sequences of Kazakhstani rabies virus isolates. Based on the results of comparative molecular genetic analysis, a conserved region of the N gene (985–1165 nt) was selected, differing from those used in previously published real-time RT-PCR test systems. The novelty of this study lies in the use of a regionally adapted approach to test system development, taking into account the genetic diversity of the rabies virus circulating in the Republic of Kazakhstan.

The analytical and diagnostic characteristics of the developed assay were compared with data from previously published real-time RT-PCR assays for RABV detection. Specifically, Panning et al. [31] developed a strain-specific real-time RT-PCR targeting the RABV nucleoprotein gene and established a 95% limit of detection (LOD) of 4.5 viral RNA copies per reaction (95% CI: 3.2–11.4), calculated using probit analysis [31]. In the present study, amplification was consistently detected at a concentration of 10 ag of recombinant plasmid DNA, corresponding to approximately 3 target copies per reaction. Thus, both values fall within the single-copy range of nucleic acid per reaction.

The linearity of the developed assay (R^2^ = 0.99) was also consistent with values reported for previously validated RT-qPCR assays. Dacheux et al. [32], in evaluating a pan-RABV RT-qPCR, obtained R^2^ values ranging from 0.900 to 0.995 depending on the RABV isolate tested; for field isolates from Thailand, France, and Argentina, R^2^ values were 0.995, 0.980, and 0.981, respectively. For an additional pan-lyssavirus RT-qPCR, R^2^ values ranged from 0.984 to 0.998 [32]. Thus, the R^2^ = 0.99 value obtained in the present study falls within the range reported for previously developed systems and indicates good amplification linearity across the concentration range studied. When assessing diagnostic performance, a combined pan-RABV/pan-lyssavirus RT-qPCR was evaluated on 211 human clinical samples, yielding an overall diagnostic sensitivity of 92.7% and specificity of 100%. Sensitivity, however, varied by clinical specimen type, reaching 91.5% for skin biopsies, 54% for saliva samples, and 100% for brain tissue samples. When the method was implemented at reference laboratories in Morocco and Cambodia, overall sensitivity and specificity were 97.7% and 100%, respectively [32].

The reproducibility of the developed assay was likewise compared with published data. In the present study, intra-assay Ct variability was low (CV = 2.67%), and when a single sample was tested across three independent analytical runs, the CV was 0.83%. In an international multicenter evaluation of the widely validated pan-lyssavirus LN34 real-time RT-PCR assay, Gigante et al. demonstrated high repeatability and reproducibility of the method: the coefficient of variation (CV) was 0.35 ± 0.06% between technical replicates, 0.72 ± 0.27% between independent extractions, and 1.33 ± 0.32% between analytical runs. Inter-laboratory variability was, as expected, higher, at 9.13 ± 2.06% [33]. Thus, the low CV values obtained in the present study indicate good repeatability and intra-laboratory reproducibility of the developed assay.

The obtained values of diagnostic sensitivity, specificity, and overall diagnostic efficiency confirm the applicability of the developed method for detecting the rabies virus and demonstrate its suitability for laboratory diagnostics. The developed test system can be used to identify rabies virus variants circulating in Kazakhstan, as well as for molecular epizootological monitoring among domestic and wild animals.

## 5. Conclusions

A comparative molecular genetic analysis of Kazakhstani rabies virus isolates was conducted. The results of the phylogenetic analysis confirm the circulation of a cosmopolitan rabies virus lineage in Kazakhstan. Based on the data obtained, a regionally adapted real-time RT-PCR test system for detecting rabies virus RNA was developed. The diagnostic sensitivity, specificity, and overall diagnostic efficacy values confirm the suitability of the method for laboratory diagnostics. The test system can be used to detect circulating rabies virus variants and conduct molecular epizootological monitoring among domestic and wild animals in the Republic of Kazakhstan.

## Figures and Tables

**Table 1:** Biological samples used to evaluate the diagnostic performance of the developed real-time RT-PCR assay.

Region	Animal Species	Number of Samples
West Kazakhstan region, KZ	Common noctule (*Nyctalus noctula*)	1
Zhambyl region, KZ	Particolored bat (*Vespertilio murinus*)	12
Turkestan region, KZ	Particolored bat (*Vespertilio murinus*)	11
Turkestan region, KZ	Common noctule (*Nyctalus noctula*)	7
Kyzylorda region, KZ	Particolored bat (*Vespertilio murinus*)	10
Zhambyl region, KZ	Lesser mouse-eared bat (*Myotis blythii*)	6
Atyrau region, KZ	Serotine bat (*Eptesicus serotinus*)	11
West Kazakhstan region, KZ	Serotine bat (*Eptesicus serotinus*)	12
Aktobe region, KZ	Serotine bat (*Eptesicus serotinus*)	13
Almaty region, KZ	Lesser mouse-eared bat (*Myotis blythii*)	7
North Kazakhstan region, KZ	Particolored bat (*Vespertilio murinus*)	16
Abai region, KZ	Serotine bat (*Eptesicus serotinus*)	4
Abai region, KZ	Cat	6
West Kazakhstan region, KZ	Cattle	5
Abai region, KZ	Cattle	6
Almaty region, KZ	Cattle	6
Aktobe region, KZ	Dog	5
Turkestan region, KZ	Jackal	6
Total		144 samples

**Table 2:** Parameters of the specific primers and probe used for the detection of rabies virus RNA.

Name	Sequence 5′→3′	Tm (°C)	GC%	Product Size (bp)
N-Rab_F	TGTTCAAYCTCATTCAYTTTGT	52	32	180
RN Probe	FAM-TGTGCTCCTCAYGAGATGTCTG-BHQ-1	70	45	
N-Rab_R	TCTTGAAGTTCTTTCTCATC	56	40	

**Table 3:** Diagnostic characteristics of the developed real-time RT-PCR test system for detecting rabies virus in relation to virus isolation as a reference method.

Indicator	Real-Time RT-PCR
Total samples	144 (100%)
True positives (TP)	19 (13.2%)
True negatives (TN)	120 (83.3%)
False positives (FP)	2 (1.4%)
False negatives (FN)	3 (2.1%)
Sensitivity (SN), 95% CI	86.4% (66.7–95.3%)
Specificity (SP), 95% CI	98.4% (94.2–99.5%)
Positive predictive value (PPV), 95% CI	90.5% (71.1–97.3%)
Negative predictive value (NPV), 95% CI	97.6% (93.1–99.2%)
Diagnostic efficiency (DE), %	96.5%

**Table 4:** Repeatability and reproducibility of the developed real-time RT-PCR assay.

Sample	1 Ct	2 Ct	3 Ct	Mean Ct ± SD	Coefficient of Variation (CV)
Repeatability (intra-assay precision)					
Rabies virus RNA	21.51	22.56	22.51	22.19 ± 0.59	2.67%
Reproducibility (inter-assay precision)					
Rabies virus RNA	22.27	22.43	22.64	22.45 ± 0.19	0.83%

**Table 5:** Detection of rabies virus by real-time RT-PCR in the tested animals.

Animal Species	Tested, n	Positive	Negative	Positive, %
Common noctule (*Nyctalus noctula*)	8	1	7	12.5
Particolored bat (*Vespertilio murinus*)	49	0	49	0
Lesser mouse-eared bat (*Myotis blythii*)	13	0	13	0
Serotine bat (*Eptesicus serotinus*)	40	0	40	0
Cat	6	3	3	50.0
Cattle	17	13	4	76.5
Dog	5	3	2	60.0
Jackal	6	2	4	33.3
Total	144	22	122	15.3

**Table 6:** Distribution of positive results across the regions of Kazakhstan.

Region	Tested	Positive	Animal Species with a Positive Result
West Kazakhstan region	18	5	common noctule, cattle
Abai region	16	7	cat, cattle
Almaty region	13	5	cattle
Aktobe region	18	3	dog
Turkestan region	24	2	jackal
Zhambyl region	18	0	-
Kyzylorda region	10	0	-
Atyrau region	11	0	-
North Kazakhstan region	16	0	-
Total	144	22	

Note: “-” positive samples were not detected.

## Data Availability

The original contributions presented in this study are included in the article. Further inquiries can be directed to the corresponding authors.
